# A biochemical hypothesis on the formation of fingerprints using a turing patterns approach

**DOI:** 10.1186/1742-4682-8-24

**Published:** 2011-06-28

**Authors:** Diego A Garzón-Alvarado, Angelica M Ramírez Martinez

**Affiliations:** 1 Associate Professor, Mechanical and Mechatronics Engineering Department, Universidad Nacional de Colombia, Engineering Modeling and Numerical Methods Group (GNUM), Bogotá, Colombia; 2 Professor, Mechanical Engineering Department, Fundación Universidad Central, Bogotá, Colombia

**Keywords:** Fingerprint, Turing pattern, numerical solution, finite element, continuum mechanics

## Abstract

**Background:**

Fingerprints represent a particular characteristic for each individual. Characteristic patterns are also formed on the palms of the hands and soles of the feet. Their origin and development is still unknown but it is believed to have a strong genetic component, although it is not the only thing determining its formation. Each fingerprint is a papillary drawing composed by papillae and rete ridges (crests). This paper proposes a phenomenological model describing fingerprint pattern formation using reaction diffusion equations with Turing space parameters.

**Results:**

Several numerical examples were solved regarding simplified finger geometries to study pattern formation. The finite element method was used for numerical solution, in conjunction with the Newton-Raphson method to approximate nonlinear partial differential equations.

**Conclusions:**

The numerical examples showed that the model could represent the formation of different types of fingerprint characteristics in each individual.

## Background

Fingerprints represent a particular characteristic for each individual [1-10]. These enable individuals to be identified through the embossed patterns formed on fingertips. Characteristic patterns are also formed on the palms of the hands and soles of the feet [1]. Their origin and development is still unknown but it is believed to have a strong genetic component, although it is not the only thing determining its formation. Each fingerprint is a papillary drawing composed by papillae and rete ridges (crests) [1-6]. These crests are epidermal ridges having unique characteristics [1].

**Figure 1:**
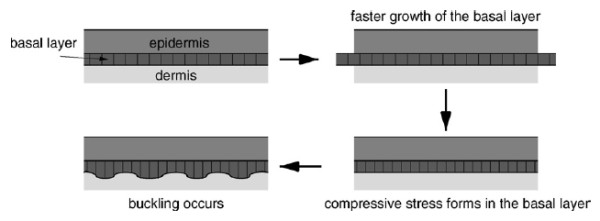
**Fingerprint formation Taken from **[7]. An explanation for the formation of grooves forming a fingerprint. The first figure on the left (top) shows the epidermis and dermis. Right: rapid growth of the basal layer. Below (right) compressive loads are generated. Left: generation of wrinkles due to mechanical loads.

Characteristic fingerprint patterns begin their formation by the sixth month of gestation [1-6]. Such formation is unchangeable until an individual's death. No two fingerprints are identical; they thus become an excellent identification tool [1,2]. Various theories have been proposed concerning fingerprint formation; among the most accepted are those that consider differential forces on the skin (mechanical theory) [1,6,7] and those having a genetic component [1,6,10]. From a mechanical point of view, it has been considered that fingerprints are produced by the interaction of nonlinear elastic forces between the dermis and epidermis [7]. This theory considers that the growth of the fingers in the embryo (dermis) is different than growth in the epidermis, resulting in folds in the skin surface [7]. Figure 1 shows a mechanical explanation for the formation of the folds that give rise to fingerprints.

**Figure 2:**
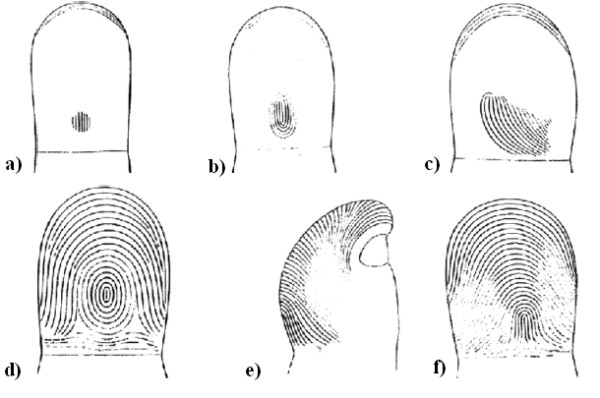
**Stages of Fingerprint formation Taken from **[9]. Fingerprint formation. a) primary formation, b) the first loop is generated, c) development. d) complete formation, e) side view, f) wear.

Fingers are separated from each other in the fetus during embryonic formation during the sixth week, generating certain asymmetries in each finger's geometry [10]. The fingertips begin to be defined from the seventh week onwards [1,10]. The first waves forming the fingerprint begin to take shape from the tenth week; these are patterns which keep growing and deform until the whole fingertip has been completed [10]. Fingerprint formation finishes at about week 19 [10]. From this time on, the fingerprints stop changing for the rest of an individual's lifetime. Figure 2 shows the stages of fingerprint formation.

Alternately to the proposal made by Kucken [7], this paper presents a hypothesis about fingerprint formation from a biochemical effect. The proposed model uses a reaction-diffusion-convection (RDC) system. Following a similar approach to that used in [11,12], a glycolysis reaction model has been used to simulate the appearance of patterns on fingertips. A solution method on three dimensional surfaces using total Lagrangian formulation is provided for resolving the reaction diffusion (RD) equations. Equations whose parameters are in the Turing space have been used for pattern formation; therefore, the patterns found are Turing patterns which are stable in time and unstable in space. Such stability is similar to that found in fingerprint formation. The model explained in [11] was used for fold growth where the formation of the folds depends on the concentration of a biochemical substance present on the surface of the skin.

## Methods

### Reaction-diffusion (RD) system

Following a biochemical approach, it was assumed that a RD system could control fingerprint pattern formation. For this purpose, an RD system was defined for two species, given by (1):(1)

where *u_1 _*and *u_2 _*were the concentrations of chemical species present in reaction terms *f *and *g, d *was the dimensionless diffusion coefficient and γ was a constant in a dimensionless system [12].

RD systems have been extensively studied to determine their behavior in different scenarios regarding parameters [12,13], geometrics [13,14] and for different biological applications [15-17]. One area that has led to developing extensive work on RD equations has been the formation of patterns which are stable in time and unstable in space [18,19]. In particular, Turing [20], in his book, "The chemical basis of morphogenesis," developed the necessary conditions for spatial pattern formation. The conditions for pattern formation determined Turing space given by the following restrictions (2):(2)

where *f*_1 _and *g*_1 _indicated the derivatives of the reaction regarding concentration variables, for example [11]. These restrictions were evaluated at the point of equilibrium by *f*(*u*_1_*, u*_2_) = *g*(*u*_1_, *u*_2_) = 0.

Equations (1) and constraints (2) led to developing the dynamic system branch of research [11,18]: Turing instability. Turing pattern theory has helped explain the formation of complex biological patterns such as the spots found on the skin of some animals [15,16] and morphogenesis problems [10]. It has also been experimentally proven that the behavior of some RD systems produce traveling wave and stable spatial patterns [21-23].

The equations used for predicting pattern formation in this paper were those for glycolysis [24], given by:(3)

where *δ *and *κ *were the model's dimensionless parameters. The steady state points were given by . Applying constraints (2) to model (3) in steady state point (*u*_1_, *u*_2_)_0 _a set of constraints was obtained. This constraint establishes the geometric site known as Turing space [24].

### Epidermis strain

The ideas suggested in [10,25,26] were used to strain the fingertip surface regarding the substances (morphogens) present in the domain; i.e. surface *S*, was strained according to its normal *N *and the amount of molecular concentration (u_2_) at each material point, therefore:(4)

where K was a constant determining growth rate.

Including the term for surface growth (equation (4)) modifies equation (1), which presented a new term taking into account the convection and dilation of the domain given by:(5)

where new term *div*(*u*_i_**v**) included convection and dilatation due to the growth of the domain, given by velocity.

The finite element method [27] was used to solve the RDC system described above in (5) and the Newton-Raphson method [28] to solve the non-linear system of partial differential equations arising from the formulation. The seed coat surface pattern growth field was imposed by solving equation (4), giving the new configuration (current) and velocity field to be included in the RD problem.

The solution of the RD equations by using the finite element method is shown below.

### Solution for RDC system

Formulating the RD system, including convective transport, could be written as (6) [24]:(6)

where *u_1 _*and *u_2 _*were the RD system's chemical variables. This equation could also be written in terms of total derivative (7) [24]:(7)

where it should be noted that

[29,30].

According to the description in [29], then the RDC system in the initial configuration, or reference Ω_0 _(with coordinates in **X(x)**), was given by the following equation, written in terms of material coordinates:(8a)(8b)

where U1 and U2 were the concentrations of each species in initial configuration Ω0, i.e. U(**X**,t) = *u(**X**(**X**,t),t)*. Besides  was the inverse of the strained gradient given by [29], *x^i^* were the current coordinates (at each instant of time) and *X^I ^*were the initial coordinates (of reference, where the calculations were to be made) [29,30].

Therefore, equation (8) gave the general weak form for (9) [27].(9)

where U was either of the two studied species (U_1 _or U_2_), W was the weighting, J was the Jacobian (and equaled the determinant for strained gradient **F**) and **C^-1 ^**was the inverse of the Cauchy-Green tensor on the right [27,28].

In the case of total Lagrangian formulation, the calculation was always done in the initial reference configuration. Therefore, the solution for system (8) and (9) began with the discretization of the variables U_1 _and U_2 _by (10) [27]:(10a)(10b)

where *nnod *was the number of nodes, **U**_1 _and **U**_2 _were the vectors containing *U_1 _*and *U_2 _*values at nodal points and superscript *h *indicated the variable discretization in finite elements. The Newton-Raphson method residue vectors were obtained by choosing weighting functions equal to shape functions (Galerkin standard) given by [27] (11):(11a)(11b)

with p = 1, ..., *nnod*, where  and  were residue vectors calculated in the new time. In turn, each position (input) of the Jacobian matrix was given by (12):(12a)(12b)(12c)(12d)

where *J *was strained gradient determinant, **C^-1 ^**was the inverse of the Cauchy-Green tensor on the right *p, s *= 1, ..., *nnod *and *I, J = 1, .., dim*, where *dim *was the dimension in which the problem was resolved. Therefore, using equations (11) and (12), the Newton-Raphson method could be implemented to solve the RD system using its material description. It should be noted that (11) and (12) were integrated in the initial configuration [29].

### Applying the velocity fields

Equation (4) was used to calculate the movement of the mesh and the velocity at which the domain was strained, integrated by Euler's method, given by [28]:(13)

where *S_t+dt _*and *S_t _*were the surface configuration in state t and t+dt. Therefore, velocity was given by (14):(14)

where the velocity term had direction and magnitude depending on the material point of surface S.

### Aspects of computational implementation

The formulation described above was used for implementing the RD model using the finite element method. It should be noted that although the surface was orientated in a 3D space, the numerical calculations were done in 2D. The normal for each element (Z') was thus found and the prime axes (X'Y') forming a parallel plane to the element plane were located. The geometry was enmeshed by using first order triangular elements with three nodes. Therefore, the calculation was simplified from a 3D system to a system which solved two-dimensional RD models at every instant of time. The relationship between the X'Y'Z 'and XYZ axes could be obtained by a transformation matrix T [29].

A program in FORTRAN was used for solving the system of equations resulting from the finite element method with the Newton-Raphson method and the following examples were solved on a Laptop having 4096 MB of RAM and 800 MHz processor speed. In all cases, the dimensionless problem was solved with random conditions around the steady state [12,24] for the RD system.

## Results

**Figure 3:**
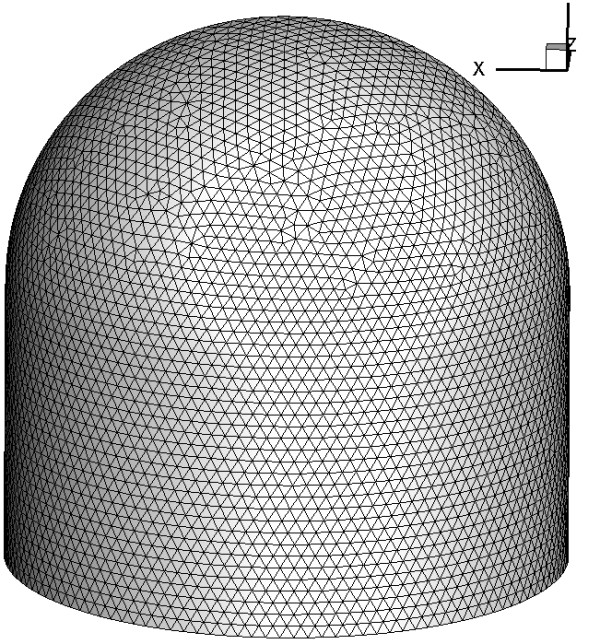
**Mesh used in the simulation**. Mesh used in developing the problem. In this figure the mesh has 5735 triangular elements and 2951 nodes.

The mesh used is shown in Figure 3. This mesh was made on a 1 cm long, 0.5 cm radius ellipsoid. The number of triangular elements was 5,735 and the number of nodes 2,951. The time step used in the simulation was dt = 2 (dimensionless). The total simulation time was t = 100.

**Figure 4:**
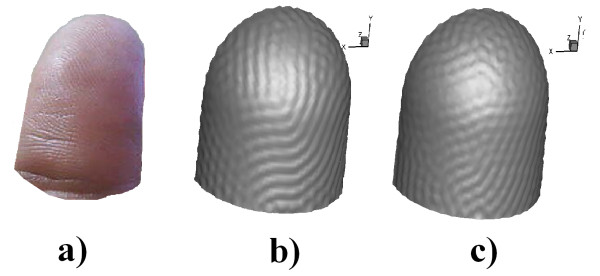
**Results of the simulation of Fingerprint a) photo of the fingerprints, b) results for parameters *d ***= **0**.08, *δ *= 1.2 and *κ *= 0.06. c) results for parameters *d *= 0.06, *δ *= 1.2 and *κ *= 0.06.

The dimensionless parameters of the RD system of glycolysis were given by *d *= 0.08, *δ *= 1.2 and *κ *= 0.06 for Figure 4a, 4b and 4c) *d *= 0.06, *δ = *1.2 and *κ *= 0.06. Therefore the steady state was given at the point of equilibrium (*u*_1_,u_2_)_0 _= (0.8,1.2), so that the initial conditions were random around steady state [12,14]. K = 0.05 in equations (4) and (13) was used for all glycolysis simulations.

Figure 4b)-4c) shows surface pattern evolution. The formation of labyrinths and blind spots in the grooves approximating the shape of the fingerprint patterns can be observed (Figure 4a). The pattern obtained was given by bands of high concentration of a chemical species, for which the domain had grown in the normal direction to the surface and hence generated its own fingerprint grooves.

**Figure 5:**
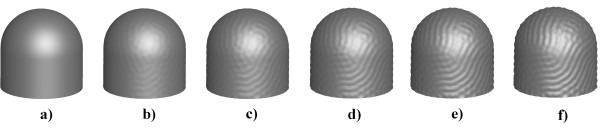
**Stages of Fingerprint formation simulation**. Different instants of time in the evolution of the folds and grooves forming the fingerprint. a) t = 0, b) t = 20, c) t = 40, d) t = 60, e) t = 80 f) t = 100. Time is dimensionless.

Figure 5 shows temporal evolution during the formation of folds and furrows on the fingertip. In 5a) shows that there was no formation whatsoever of folds. In b), small bumps began to form, in the entire domain, which continued to grow and form the grooves, as shown in Figure 5f).

## Discussion and conclusions

This paper has presented a phenomenological model based on RD equations to predict the formation of rough patterns on the tips of the fingers, known as fingerprints. The application of the RD models with Turing space parameters is an area of constant work and controversy in biology [31,32] and has attracted recent interest due to the work of Sick *et al*., [32] confirming the validity of RD equations in a model of the appearance of the hair follicle. From this point of view, the work developed in this article has illustrated RD equation validity for representing complex biological patterns, such as patterns formed in fingerprints.

This paper proposes the existence of a reactive system (activator-inhibitor) on the skin surface giving an explanation for the patterns found. The high stability of the emergence of the patterns can also be explained, i.e. the repetition of the patterns was due to a specialized biochemical system allowing the formation of wrinkles in the fingerprints and skin pigmentation.

The formulation of a system of RD equations acting under domain strain was programmed to test this hypothesis. Continuum mechanics thus led to the general form of the RD equations in two- and three-dimensions on domains presenting strain. The resulting equations were similar to those shown in [33], where major simplifications were carried out on field dilatation. The RD system was solved by the finite element method, using a Newton-Raphson approach to solve the nonlinear problem. This allowed longer time steps and obtaining solutions closer to reality. The results showed that RD equations have continuously changing patterns.

Additionally, it should be noted that the results obtained with the RD mathematical model was based on assumptions and simplifications that should be discussed for future models.

The model was based on the assumption of a tightly coupled biochemical system (non-linear) between an activator and an inhibitor generating Turing patterns. As far as the authors know, this assumption has not been tested experimentally, so the model is a hypothesis to be tested in future research. It is also feasible, as in other biological models (see [7]), that there were a large number of chemical factors (morphogens) involved, interacting to form superficial patterns found in the fingers. In the case of patterns with superficial roughness, the biochemical system could also interact with its own mechanical growth factors. Therefore, determining the exact influence of each biochemical and mechanical factor on the formation of surface patterns becomes an experimental challenge that will reveal the morphogenesis of fingerprints.

## Competing interests

The authors declare that they have no competing interests.

## Authors' contributions

The work was made by equal parts, in manuscript, modelling and numerical simulation. All authors read and approved the final manuscript.
